# 

**Published:** 1896-07

**Authors:** 


					﻿Vol. XVI.
JULY, 1896.
NO. 7.
THE
Pomœopathic pn Y^iciAp,
A Monthly Journal of Homoeopathic Materia Medica
and Clinical Medicine.
** He is a freeman whom truth makes free, and all are slaves beside."
"Seek the Truth: come whence it may, cost what it will."
EDITED AND PUBLISHED BY
WALTER M. JAMES, M. D.
PHILADELPHIA :
USTo. 1231 LOCUST STREET.
LONDON :
ALFRED HEATH & CO., 8ole Agents fob', Gbeat Britain,
114 Ebury Street, Eaton Square.
•Yearly Subscription, $2.50, invariably in advance. Sinyle numbers, 25 els.
Entered at the Philadelphia Poat-OEce as Second-Claea Mall Matter.
				

